# Synthetic Strategies for Peroxide Ring Construction in Artemisinin

**DOI:** 10.3390/molecules22010117

**Published:** 2017-01-11

**Authors:** Vera A. Vil’, Ivan A. Yaremenko, Alexey I. Ilovaisky, Alexander O. Terent’ev

**Affiliations:** 1 N. D. Zelinsky Institute of Organic Chemistry, Russian Academy of Sciences, 47 Leninsky Prospekt, Moscow 119991, Russia; vil@ioc.ac.ru (V.A.V.); yaremenko@ioc.ac.ru (I.A.Y.); ilov@ioc.ac.ru (A.I.I.); 2 Faculty of Chemical and Pharmaceutical Technology and Biomedical Products, D. I. Mendeleev University of Chemical Technology of Russia, 9 Miusskaya Square, Moscow 125047, Russia; 3 All-Russian Research Institute for Phytopathology, 143050 B. Vyazyomy, Moscow Region, Russia

**Keywords:** artemisinin, artemisinic acid, organic peroxide, peroxidation, cycles

## Abstract

The present review summarizes publications on the artemisinin peroxide fragment synthesis from 1983 to 2016. The data are classified according to the structures of a precursor used in the key peroxidation step of artemisinin peroxide cycle synthesis. The first part of the review comprises the construction of artemisinin peroxide fragment in total syntheses, in which peroxide artemisinin ring resulted from reactions of unsaturated keto derivatives with singlet oxygen or ozone. In the second part, the methods of artemisinin synthesis based on transformations of dihydroartemisinic acid are highlighted.

## 1. Introduction

In accordance with World Health Organization reports, malaria is one of the most widespread diseases, with 149–303 million of new malaria cases and 438,000 malaria deaths registered in 2015 all over the world. The largest number of new infection cases occur in the African Region (88%), the second one in Southeast Asia (10%), and the third in the Eastern Mediterranean (2%) [1].

Malaria is an infection caused by unicellular organisms of the *Plasmodium* type parasites. The growth and development of the parasites in red blood cells determine the pathology of malaria. In most cases, the disease is initiated by *Plasmodium falciparum* infection, although *P. vivax*, *P. ovale*, *P. malariae*, and *P. knowlesi* infection also cause malaria [2,3].

Traditionally, some drugs, such as quinine, chloroquine, mefloquine, doxycycline, and other antiparasitic ones, have been used for malaria treatment. However, the development of antibiotic resistance of *Plasmodium* significantly reduced the efficacy of therapy [4,5].

Much effort has been directed toward the treatment and prevention of malaria; as a result, the incidence of malaria (new cases of infection) reduced by 37% worldwide and by 42% in the African Region from 2000 to 2015. Over the same period, the rate of malaria deaths decreased by 60% worldwide and by 66% in the African Region [1]. Such impressive results have been achieved due to a complex of measures: insecticide-treated mosquito nets (ITNs), indoor residual spraying (IRS), rapid diagnostic testing (RDTs), and mainly artemisinin-based combination therapy (ACT) [6,7]. Artemisinin-based combination therapy (ACT) is highly efficient against malaria caused by *P. falciparum*, which is the most abundant and pathogenic type of *Plasmodium* for humans [8].

Artemisinin (Qinghaosu) (**1**) is a natural peroxide with high antimalarial activity [9,10]. It has been playing a key role in malaria treatment over the past several decades. Artemisinin was isolated from *Artemisia annua* for the first time in 1971 in the context of the scientific program “Project 523”, initiated by the Chinese government in 1967 [11,12,13]. The Nobel Prize in Physiology or Medicine 2015 was awarded to Chinese pharmaceutical chemist Tu Youyou “for her discoveries concerning a novel therapy against Malaria” [14,15,16].

The availability of artemisinin-based drugs is largely limited by their high cost [17,18]. Its isolation from *Artemisia annua* is still the main way for artemisinin production [19,20,21]. Full cycle time is 12–18 months, while overall yield of isolated artemisinin is usually less than 1 wt % [22,23].

**Figure 1:**
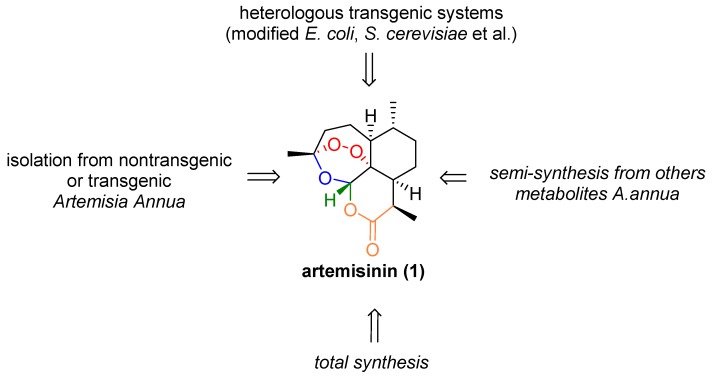
Main strategies for artemisinin production.

As a result, the production of a sufficient amount of artemisinin with the use of only an isolation method remains an extremely challenging task. The problem of the sustainable manufacturing of artemisinin can be solved through a nontransgenic method [24] (selection, variation of nutrients and ambient conditions, and the use of test-tube cultures [25]), the use of transgenic *A. annua* [26], heterologous transgenic systems [27,28] (introduction of genes encoding the artemisinin biosynthesis into others cultures), semi-synthetic approaches based on available relative structures [29] and total synthesis [30] (Figure 1).

Synthetic strategies for artemisinin production have been reported in several reviews [31,32,33,34,35]. The interdisciplinary approaches to the preparation of artemisinin were demonstrated by Abdin et al. [36] and Corsello et al. [37]. Semi-synthetic methods for artemisinin (**1**) production from artemisinic (**3**) and dihydroartemisinic (**4**) acids, more available metabolites of *Artemisia annua*, were shown in two other reviews [38,39].

1,2,4-Trioxolane cycle spiro-conjugated with lactone is the key pharmacophore fragment in artemisinin. The construction of this fragment is the most difficult stage both in synthetic and biological approaches to artemisinin production. The construction of peroxide fragment of artemisinin is carried out mainly in final synthetic steps with the use of one-pot peroxidation/cyclization reactions.

**Scheme 1:**
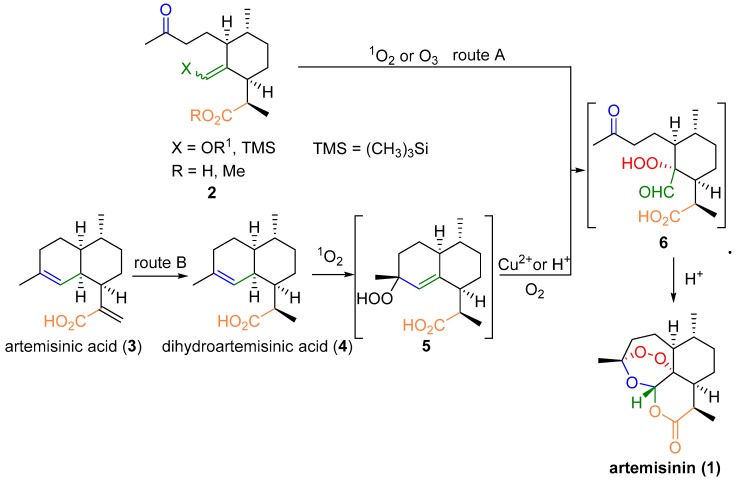
Peroxidation steps based on the formation of hydroperoxides in synthetic approaches to artemisinin (**1**).

The present review is devoted to the strategies for construction of artemisinin peroxide fragment in synthetic and semi-synthetic approaches (Scheme 1). According to the published data, unsaturated keto derivatives **2** (route A) or dihydroartemisinic acid (**4**) (route B) serve as the precursor substance on final peroxidation/cyclization step in artemisinin synthesis. Synthetic methods where dihydroartemisinic acid (**4**) obtained from artemisinic acid (**3**) is directly oxidized into artemisinin (**1**) (route B) deserve special attention, due to the fact that these approaches form the basis of modern methods of artemisinin production [40]. In 2013, the Sanofi company launched the semi-synthetic artemisinin manufacturing based on photooxidation of dihydroartemisinic acid (**4**) [41].

In the peroxidation step, the unsaturated keto derivative **2** initially forms the hydroperoxyaldehyde **6**, cyclizing into artemisinin (**1**) under the action of acids. Peroxidation of dihydroartemisinic acid (**4**) starts from the formation of intermediate **5**, which was converted into artemisinin (**1**) via compound **6**. Consequently, in the present review, the data are primarily classified according to the structures of final precursors **2** and **4** and secondly according to the peroxidation methods.

## 2. Construction of Artemisinin Peroxide Fragment in Total Synthesis

### 2.1. Using Singlet Oxygen

**Scheme 2:**
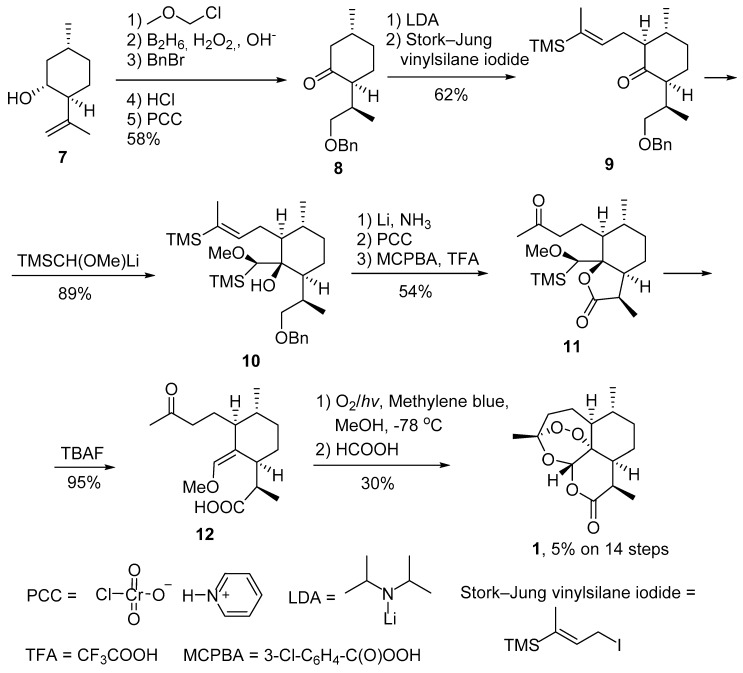
Total synthesis of artemisinin by Schmid and Hofheinz.

In 1983, Schmid and Hofheinz presented the first example of artemisinin (**1**) total synthesis (Scheme 2) [30]. The available terpene alcohol, (−)-isopulegol (**7**), was used as a starting compound. The ketone **8** was synthesized from (−)-isopulegol (**7**) in five steps with a 58% yield. Later, it was alkylated by Stork–Jung vinylsilane iodide with the formation of silylated ketone **9** with a 62% yield. The key intermediate **12** was synthesized from silylated ketone **9** in four steps. After the treatment of ketone **9** by excess of TMSCH(OMe)Li, the major diastereomer **10** was isolated with an 89% yield (ratio of diastereomers 8:1). The lactone **11** prepared by successive debenzylation and oxidation of alcohol **10** was transformed into enol ether **12** by TBAF (tetra-*n*-butylammonium fluoride). Ene reaction of enol ether **12** with ^1^O_2_ and subsequent acid-catalyzed cyclization of obtained hydroperoxide led to desired artemisinin (**1**) with a 30% yield. The total yield was 5% in 14 steps.

**Scheme 3:**
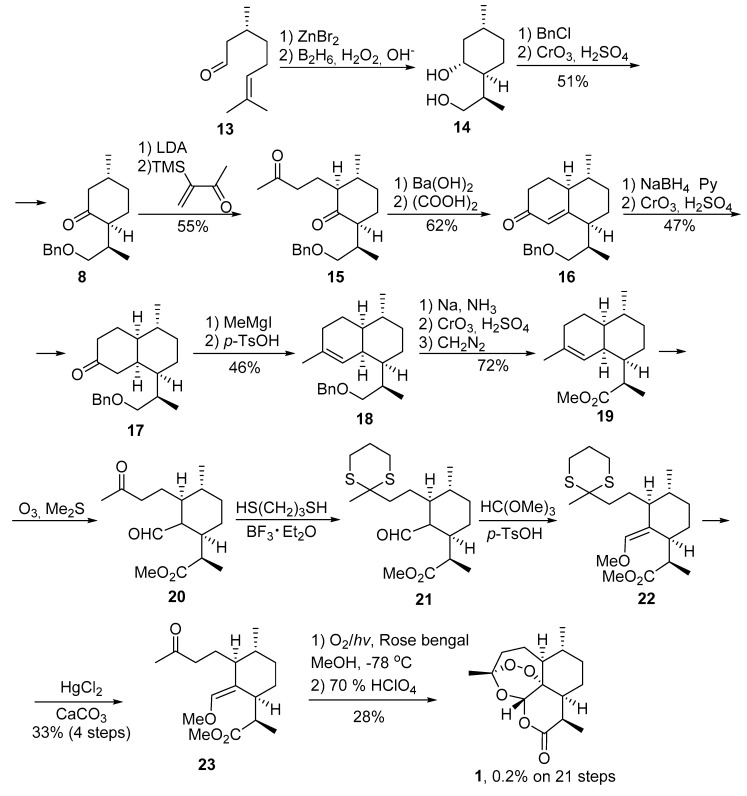
Total synthesis of artemisinin by Zhou and co-workers.

Zhou et al. demonstrated the second example of artemisinin total synthesis in 1986 (Scheme 3) [42]. *R*-(+)-Citronellal (**13**), chosen as a starting material, was initially transformed into dihydroxy compound **14**. The benzylation and subsequent oxidation of **14** resulted in ketone **8** with a 51% yield. The reaction of deprotonated ketone **8** with silylated vinyl ketone afforded 1,5-diketone **15**, which was transformed into unsaturated ketone **16** with a 62% yield. The reduction of unsaturated ketone **16** followed by oxidation by chromium trioxide led to ketone **17** with a 47% yield. The addition of MeMgI to ketone **17** followed by dehydration afforded the mixture of isomers from which the unsaturated product **18** was isolated with a 46% yield. It was transformed into methyl ester of dihydroartemisinic acid (**19**) in three steps with a 72% yield. Ozonolysis of **19** afforded ketoaldehyde **20**. The ketone group of **20** was protected by 1,3-propanedithiol with the formation of intermediate **21**, which was converted into the enol ether **22** with the use of trimethyl orthoformate. The deprotection of **22** afforded enol ether **23**. The photooxidation of enol ether **23** in the presence of oxygen and Rose Bengal following by the treatment of 70% HClO_4_ resulted in artemisinin (**1**) with a yield of 0.2% in 21 steps.

**Scheme 4:**
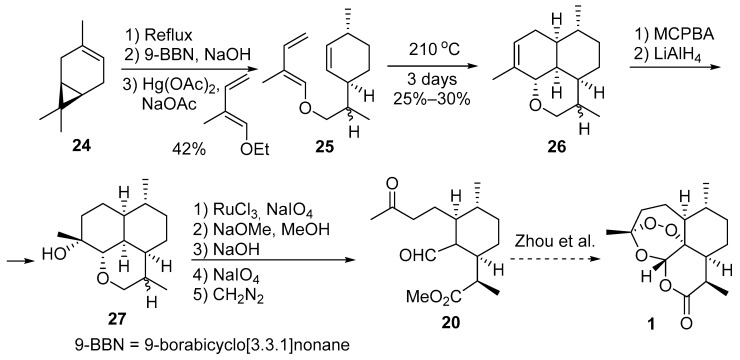
The synthesis of ketoaldehyde **20** through the Diels–Alder approach.

A few years later, Ravindranathan et al. reported the approach to aldehyde **20**, which reduced the synthetic pathway from 15 to 8 steps [43]. The synthesis of key precursor **20** was based on an intramolecular Diels–Alder reaction of triene **25** prepared from (+)-3-carene (**24**) in three steps (Scheme 4). The triene **25** was cyclized into unsaturated ether **26** under high temperature conditions. The epoxidation of **26** followed by reduction by LiAlH_4_ led to alcohol **27**, which was transformed into key precursor **20** in five steps.

**Scheme 5:**
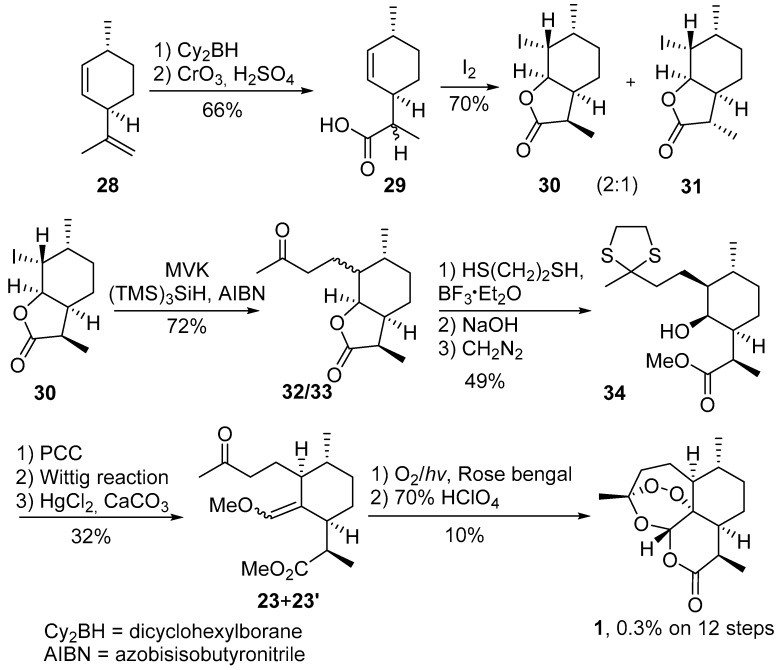
Total synthesis of artemisinin by Yadav and co-workers (2003).

Yadav and colleagues used (+)-isolimonene (**28**) obtained by (+)-2-carene heating as a starting reagent for the synthesis of artemisinin. Hydroboration with subsequent oxidation of (+)-isolimonene (**28**) resulted in acid **29** (Scheme 5) [44]. Iodolactonization of acid **29** led to the separable mixture of iodolactones **30** and **31**. The intermolecular radical reaction of iodolactone **30** with methyl vinyl ketone (MVK) resulted in the formation of diastereomers **32**/**33** mixture, which can be separable by the introduction of thioacetal fragment. Methyl ester **34** was prepared by saponification and consequent methylation of thioacetal-derivative. The oxidation of hydroxyl-group, the introduction of methoxymethyl fragment, and the removal of the thioacetal group led to the mixture of enol ether **23** isomers with a 32% yield. The construction of bicyclic peroxide fragment of artemisinin is a two-step process. The first step is photooxidation of the mixture of enol ether **23** isomers by ^1^O_2_, and the second one is acid-catalyzed cyclization. The total yield of artemisinin (**1**) was 0.3% in 12 steps.

**Scheme 6:**
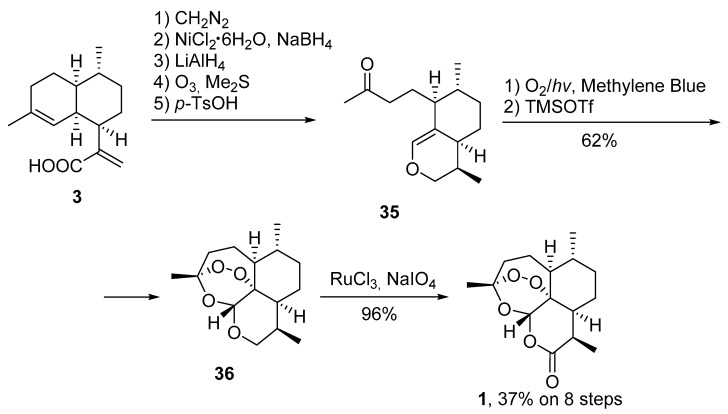
Synthesis of artemisinin from artemisinic acid (**3**) via enol ether **35**.

The synthesis of artemisinin (**1**) based on artemisinic acid (**3**) with a total yield of 37% via cyclic enol ether **35** was reported (Scheme 6) [45]. Photooxidation of **35** using ^1^O_2_ followed by the treatment with TMSOTf (trimethylsilyl trifluoromethanesulfonate), resulted in deoxoartemisinin (**36**). Oxidation of **36** by the RuC1_3_–NaIO_4_ system afforded **1** with a 96% yield.

**Scheme 7:**
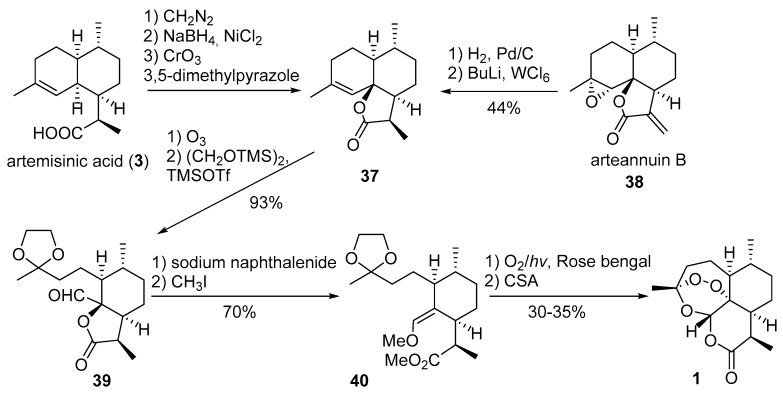
The synthesis of artemisinin by Lansbury and Nowak.

An efficient partial synthesis of artemisinin (**1**) starting from artemisinic acid or arteannuin B (**38**) through enol ether **40** was reported by Lansbury and Nowak (Scheme 7) [46]. Artemisinic acid or arteannuin B were converted to lactone **37**. Reduction of double bond of arteannuin B (**38**) followed by deoxygenation of formed saturated epoxide resulted in lactone (**37**) with a 44% yield. Ozonolysis of **37** and protection of obtained carbonyl group led to aldehyde **39** that was transformed into enol ether **40** by the cleavage of lactone ring with subsequent methylation. The construction of artemisinin peroxide fragment was realized by photooxidation with ^1^O_2_ followed by camphorsulfonic acid (CSA)-catalyzed cyclization. The total yield of artemisinin (**1**) from arteannuin B (**38**) was 10% in eight steps. Later, the authors proposed an alternative route from arteannuin B (**38**) to artemisinin (**1**) in six steps with a 5% yield [47].

**Scheme 8:**
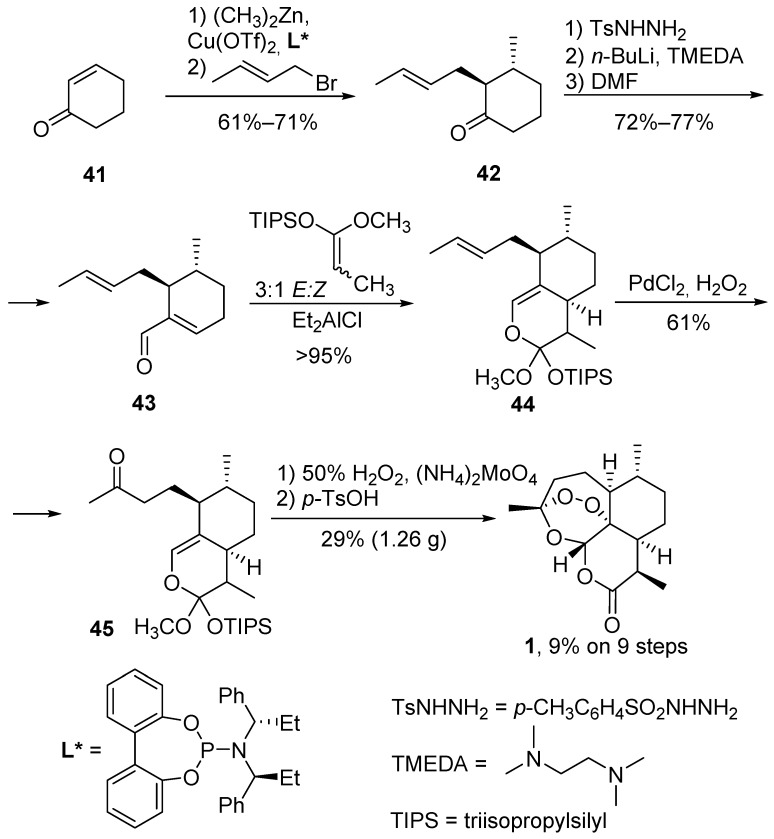
The gram scale total synthesis of artemisinin by Cook and co-workers.

A cost-effective total synthesis of artemisinin with a total yield of 9% in nine steps was reported by Cook and colleagues. They conducted a gram scale synthesis from cyclohexenone (**41**)—a widely available starting material (Scheme 8) [48]. The synthesis of α,β-unsaturated aldehyde **43** was performed by successive Michael-type methylation, allylation with the formation of ketone **42**, and Shapiro-process. The unusual [4 + 2] reaction between α,β-unsaturated aldehyde **43** and a silyl ketene acetal provided ortho ester **44**. The feature of proposed total synthesis of artemisinin lies in using of selective oxidation reactions in two final steps. The mild Wacker-type oxidation of internal olefin of **44** afforded enol olefin **45**. The final oxidative rearrangement of the latter by singlet oxygen from the decomposition of H_2_O_2_ by ammonium molybdate resulted in artemisinin (**1**).

### 2.2. Other Methods

**Scheme 9:**
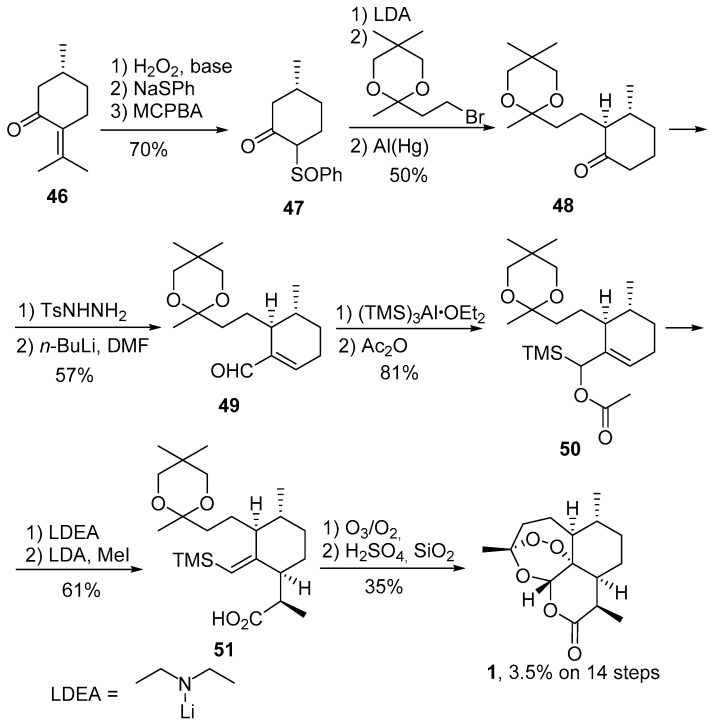
The total synthesis of artemisinin by Avery and co-workers (1992).

Avery and colleagues developed quite a different precursor for the construction of bicyclic peroxide artemisinin fragment—vinyl silane **51** (Scheme 9) [49,50]. The synthesis started from the transformation of (*R*)-(+)-pulegone (**46**) into sulfoxide **47** with a 70% yield. The alkylation of sulfoxide **47** with LDA and 1,3-dioxane-derivative bromide followed by desulfurization with an aluminum amalgam afforded the ketone **48** with a 50% yield. The two-step Shapiro reaction converted ketone **48** into homologous aldehyde **49**. The silylation and acylation of aldehyde **49** produced silane **50**, which was transformed into vinyl silane **51** via Ireland–Claisen rearrangement. The reaction of vinyl silane **51** (the step of removing of acetal protecting group was in the first version of synthesis [49]) with ozone followed by the rearrangement of unstable peroxide intermediate provided artemisinin (**1**) with a 35% yield (the total yield was 3.5% in 14 steps).

**Scheme 10:**
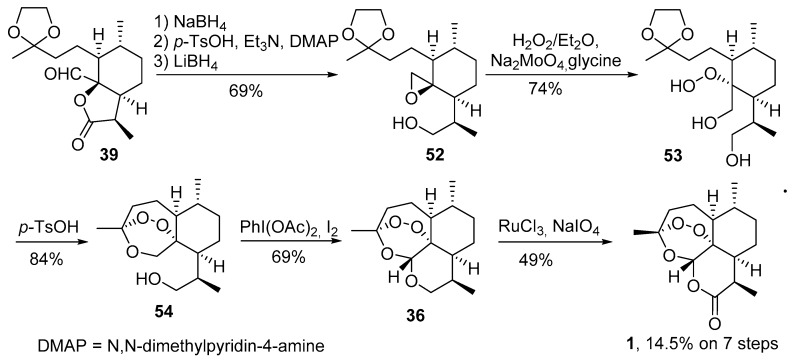
The hydrogen peroxide-based access to artemisinin.

Wu and colleagues reported the hydrogen peroxide-based access to artemisinin (**1**) with an overall yield of 14.5% in seven steps starting from aldehyde **39** converted into spiro epoxide **52** (Scheme 10) [51]. Addition of H_2_O_2_ to the highly hindered quaternary C-12a in precursor **52** was achieved through a facile perhydrolysis of a spiro epoxy ring with the help of molybdenum species (prepared from Na_2_MoO_4_ and glycine). Resulting β-hydroxyhydroperoxide **53** was further cyclized by *p*-TsOH with the formation of peroxide **54**, followed by oxidation, which resulted in ketal **36**. The artemisinin (**1**) was prepared by the oxidation of **36** by the RuCl_3_/NaIO_4_ system.

## 3. Artemisinin Synthesis Based on Dihydroartemisinic Acid

### 3.1. Preparation of Dihydroartemisinic Acid

Currently, semi-synthetic methods of artemisinin (**1**) synthesis from available biosynthetic precursors such as artemisinic acid (**3**) have attracted an increasing amount of attention as cost-effective, environmentally friendly, and high-quality approaches to the production of artemisinin from reliable sources [40].

Artemisinic acid, also called arteannuic acid (**3**), is 8–10-fold more abundant in *A. annua* than **1** [39] and can be isolated without chromatography [52]. The developed biosynthetic methods allow for the production of artemisinic acid on a large scale with the use of other organisms [53,54,55]. In 2006, the fermentation process with engineered yeast to produce high titers of artemisinic acid was developed in Amyris Inc. and the University of California [56]. In 2013, Paddon, Newman, and co-workers reported the method for biological production of artemisinic acid with the help of genetically modified strains of *Saccharomyces cerevisiae* (baker’s yeast). This development avoids the increase in fermentation titers of produced artemisinic acid to 25 g/L [57].

One of the most critical steps during the production of artemisinin is the regio- and diastereoselective reduction of artemisinic acid (**3**) to diastereomeric dihydroartemisinic acid (**4**). The most perspective method in the context of industry is the reduction by hydrogen (22–46 atm) on Rh-based catalysts—(Ph_3_P)RhCl_2_ [57], RuCl_2_[(*R*)-DTBM-Segphos](DMF)_2_ (the full name of ligand (*R*)-DTBM-Segphos is (*R*)-(−)-5,5′-Bis[di(3,5-di-tert-butyl-4-methoxyphenyl) phosphino]-4,4′-bi- 1,3-benzodioxole) [58], the yield of dihydroartemisinic acid (**4**) was > 98% and 99%, respectively. In addition, NaBH_4_/NiCl_2_ [59] (98% yield) and LiBH_4_/NiCl_2_ [60] (quantitative yield) were used for the reduction of artemisinic acid. An attractive method toward the diastereoselective synthesis of dihydroartemisinic acid (**4**) from artemisinic acid (**3**) was realized with use of diimide as a reducing agent (generated by the reaction of hydrazine monohydrate and oxygen) in the pilot plant scale-up with a yield of >90% [61,62,63]. Moreover, dihydroartemisinic acid (**4**) or its methyl ester were prepared in 17 steps from (−)-β-pinene [64], in 9 steps from (−)-isopulegol [65], and in 8 steps from (*R*)-(+)-citronellal [66].

### 3.2. Transformation of Dihydroartemisinic Acid into Artemisinin via Hydroperoxidation and Rearrangements

**Scheme 11:**
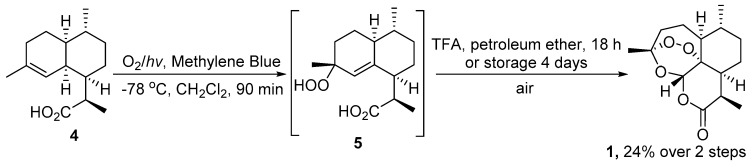
The transformation of dihydroartemisinic acid (**4**) into artemisinin (**1**).

The first example of two-step conversion of dihydroartemisinic acid (**4**) into artemisinin (**1**) was reported by Roth and Acton in 1989 [29]. Dihydroartemisinic acid (**4**) was photooxidized into hydroperoxide **5**, which in situ rearranged into artemisinin (**1**) by action of trifluoroacetic acid (TFA) (Scheme 11).

**Scheme 12:**
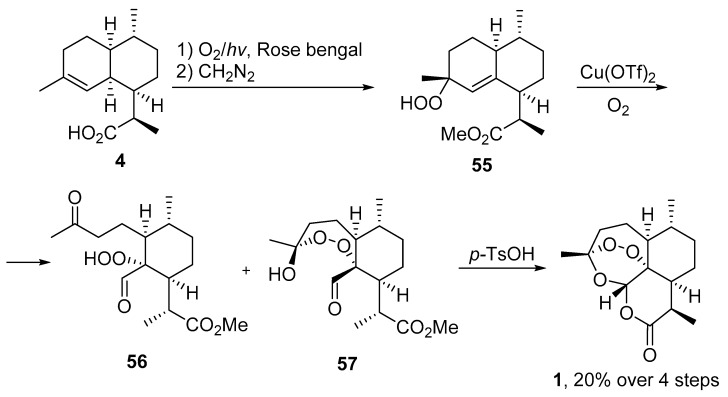
The transformation of dihydroartemisinic acid (**4**) into artemisinin (**1**) via hydroperoxyester **55**.

Photooxygenation of dihydroartemisinic acid (**4**) with subsequent CH_2_N_2_ treatment provided corresponding ester **55** which was converted into peroxides **56** and **57** with a catalytic amount of Cu(OTf)_2_ (Scheme 12) [67]. The artemisinin (**1**) was prepared by *p*-TsOH-catalyzed cyclization of the crude mixture of peroxides **56** and **57**. Additional methylation step did not significantly affect the yield of artemisinin. The total artemisinin (**1**) yield was 20% in four steps.

Later, many attempts have been made to in situ transformation of dihydroartemisinic acid (**4**) to artemisinin (**1**) (Table 1); however, the yield of artemisinin (**1**) did not exceed 30%.

**Scheme 13:**
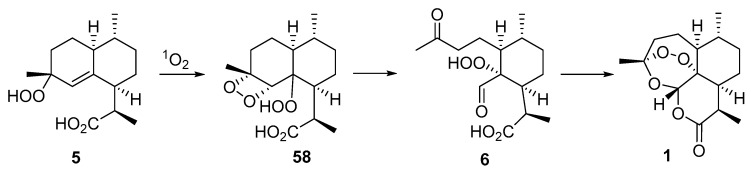
The mechanistic possibilities for the conversion of artemisinic acid into artemisinin.

The mechanism of transformation of dihydroartemisinic acid (**4**) into artemisinin (**1**) was thoroughly studied [68,69,70,71,72,73]. The first step is peroxidation of **4** to form **5**. Initially, it was suggested that conversion of hydroperoxide **5** into artemisinin (**1**) mainly proceeds through [2 + 2] cycloaddition of singlet oxygen to **5**, with the formation of intermediate **58** followed by oxidation to intermediate **6** (Scheme 13) [74].

**Scheme 14:**
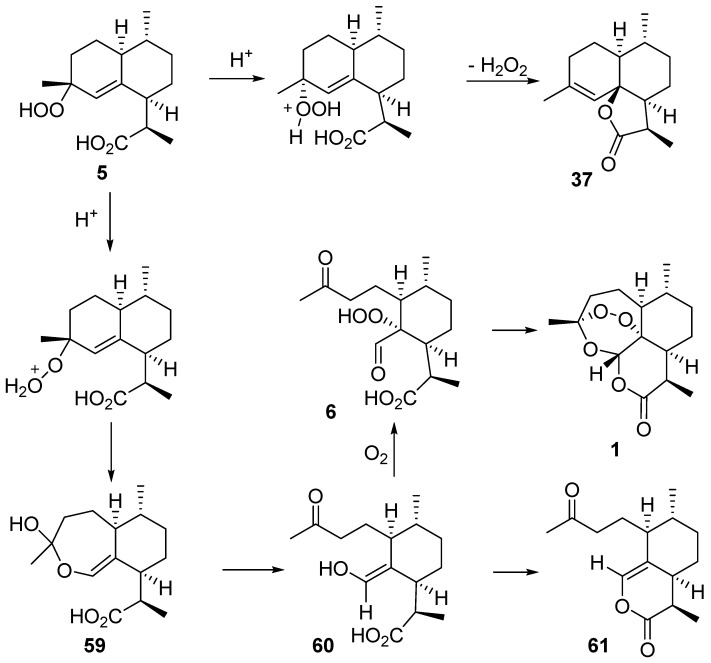
Acid-catalyzed reaction steps in the formation of artemisinin and side products.

Later, it was shown that the key steps involve terminal protonation of hydroperoxide **5**, Hock cleavage via intermediate **59**, and reaction of enol **60** with triplet oxygen to form hydroperoxide **6**, which is cyclized to artemisinin (**1**) (Scheme 14) [75]. The formation of possible by-products, such as five-membered lactone dihydro-epi-deoxyarteannuin B (**37**) and enol ester **61**, was minimized by TFA catalysis.

**Scheme 15:**
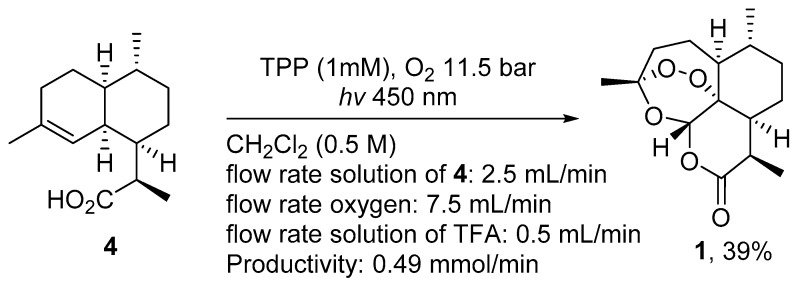
Reaction conditions for continuous flow synthesis of artemisinin.

A continuous-flow process for the synthesis of artemisinin (**1**) from dihydroartemisinic acid (**4**), which does not require isolation and purification of intermediates, was proposed (Scheme 15) [76]. The key step is a photochemical transformation involving a singlet-oxygen induced ene reaction, Hock cleavage, and the addition of triplet oxygen, followed by cyclization with the formation of an endoperoxide group. Tetraphenylporphyrin (TPP) was used as photosensitizer. Isolated yield of artemisinin (**1**) was 39% (1.36 g).

Later, the authors succeeded in optimizing this one-pot photochemical continuous-flow method for the synthesis of artemisinin from dihydroartemisinic acid (**4**). The optimization of reaction conditions was conducted (LED lamp emitting at 420 nm, temperature, solvent, TFA concentration); as a result, the isolated yield of artemisinin (**1**) increased to 57% [75]. 

**Scheme 16:**
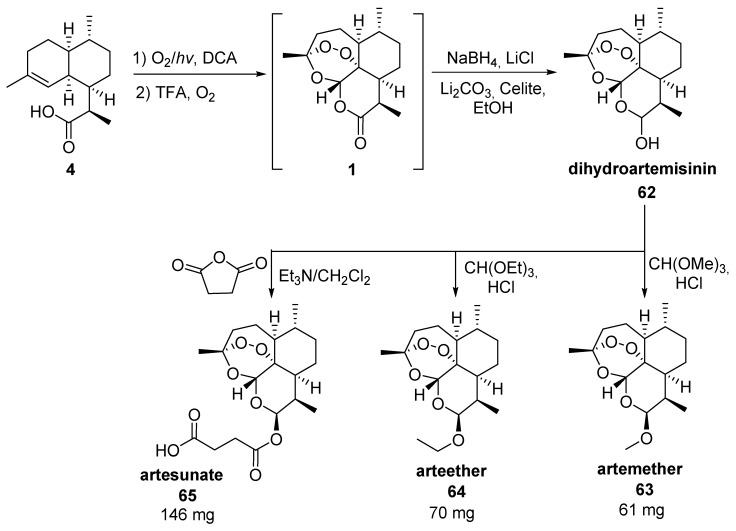
Flow-synthesis of artemisinin derivatives—dihydroartemisinin (**62**), artemether (**63**), arteether (**64**), and artesunate (**65**).

The continuous synthesis of derivatives of artemisinin: dihydroartemisinin **62**, artemether **63**, arteether **64**, and artesunate **65** was also realized (Scheme 16) [77]. The system consists of three linkage modules for photooxidation/cyclization (9,10-dicyanoanthracene (DCA) was chosen as photosensitizer), reduction, and derivatization steps. The purification of crude products was carried out by column chromatography in fourth module.

**Scheme 17:**
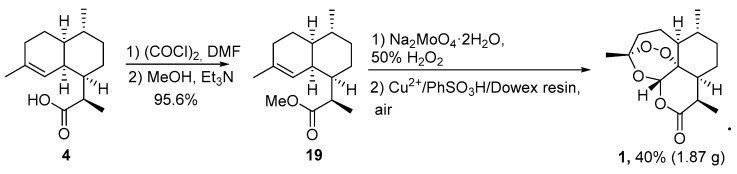
Conversion of dihydroartemisinic acid to artemisinin using a chemical source of singlet oxygen.

The Paddon group showed that chemical conversion of dihydroartemisinic acid (**4**) into artemisinin (**1**) can be performed without specific photochemical equipment (Scheme 17) [57]. The methylation of dihydroartemisinic acid (**4**) afforded methyl ester **19**, which was oxidized by singlet oxygen, generated through the disproportionation of concentrated H_2_O_2_ under the action of molybdate catalyst.

**Scheme 18:**
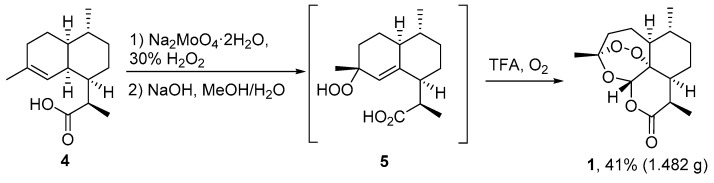
The gram-scale synthesis of artemisinin by Wu.

Using the same Na_2_MoO_4_/H_2_O_2_ protocol, Yikang Wu also realized the gram-scale synthesis of artemisinin (**1**) without any special equipment (Scheme 18) [78]. Ene reaction of dihydroartemisinic acid (**4**) with singlet oxygen afforded intermediate **5**, which was rearranged into artemisinin (**1**) by action of TFA and oxygen.

**Figure 2:**
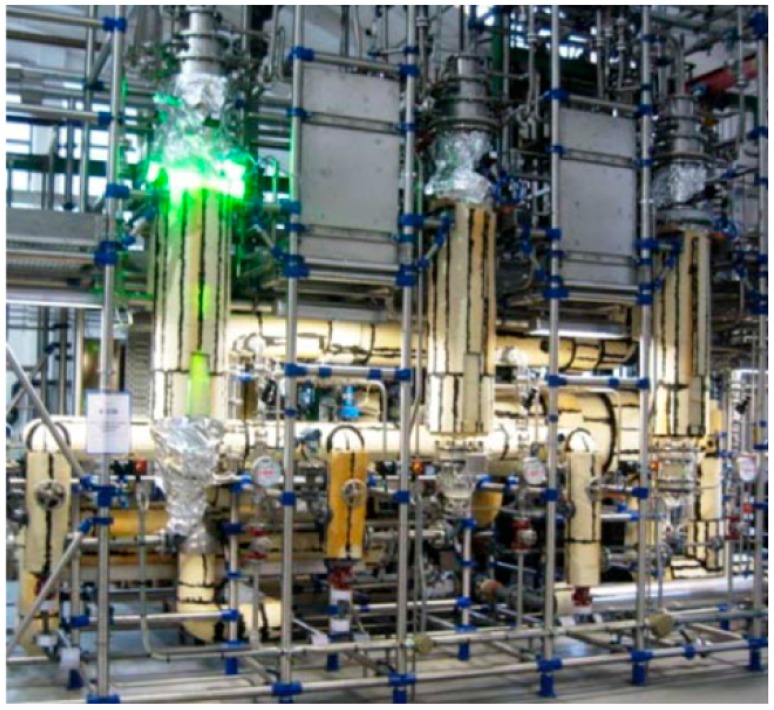
Photooxidation process in semi-synthetic artemisinin (**1**) production by Sanofi (Reprinted with permission from [58] Copyright 2016 American Chemical Society).

**Scheme 19:**
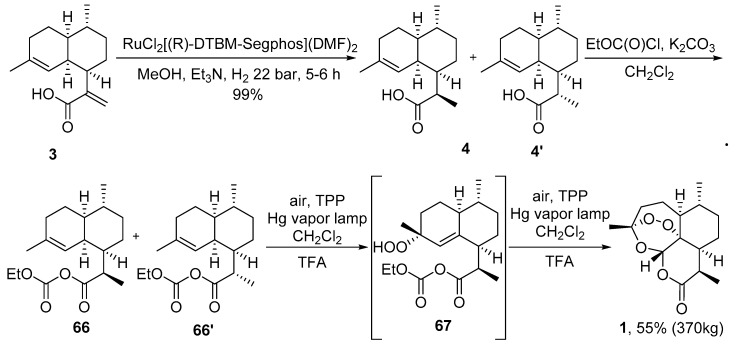
The production of semi-synthetic artemisinin (**1**) by Sanofi.

Sanofi company developed the large-scale process of artemisinin (**1**) production from artemisinic acid (**3**) using diastereoselective hydrogenation and photooxidation (Scheme 19, Figure 2) [58]. At 22 bar of H_2_, a mixture of enantiomers **4** and **4′** was obtained with the help of RuCl_2_[(*R*)-DTBM-Segphos](DMF)_2_ catalyst. The activated esters **66** and **66′** (mixed anhydride) were obtained from **4** and **4′** with the use of ethyl chloroformate/potassium carbonate. They were expected to facilitate the final ring closure. Mixed anhydrides were introduced to the Schenck ene reaction using tetraphenylporphyrin (TPP) in CH_2_Cl_2_ as a sensitizer. Cascade of several chemical steps (Hock cleavage, additional oxygenation, ring closure) initiated by TFA resulted in a high overall yield (55%) of isolated artemisinin (**1**) (370 kg) from artemisinic acid (**3**) used as the starting material (Scheme 19).

**Scheme 20:**
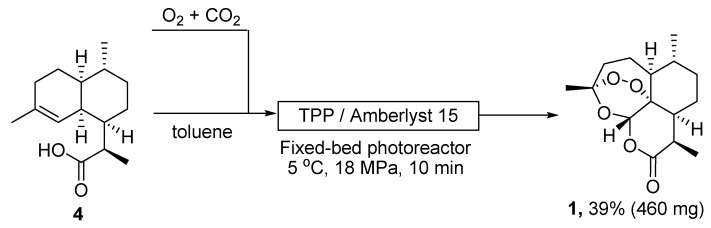
Synthesis artemisinin (**1**) with use of dual solid catalyst.

Rossen, Poliakoff, and George with colleagues reported two green chemistry approaches to artemisinin (**1**) from dihydroartemisinic acid (**4**) [79]. The first strategy lies in the use of liquid CO_2_ as a solvent and photocatalyst (commercially available porphyrins—TPP and TPFPP) immobilized onto the sulfonated ion-exchange resin Amberlyst-15; 460 mg of artemisinin (**1**) was isolated (Scheme 20).

**Scheme 21:**
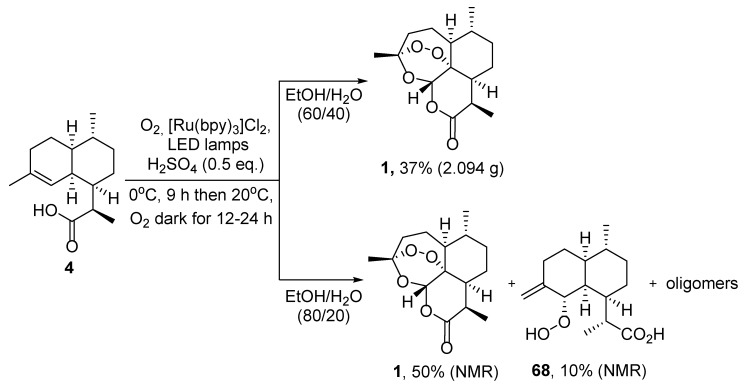
The synthesis of artemisinin (**1**) in an aqueous condition.

The second strategy [79] is based on an application of alcohol/water mixtures. The major advance of this method is the possibility of recycling of solvents, photocatalyst (tris(bipyridine)ruthenium(II) chloride), and aqueous acid (Scheme 21).

The isolated yield of artemisinin (**1**) after three cycles is 37% (2.094 g). The authors [79] attempted to explain the modest yield of artemisinin (**1**). In slightly changed reaction conditions (Scheme 21, the lower equation), artemisinin (**1**) (50% NMR yield) and the stable minor hydroperoxide **68** (10% NMR yield) were detected and isolated. Hydroperoxide **68** is likely formed as a by-product in the ene reaction of dihydroartemisinic acid (**4**) with singlet oxygen. After column chromatography of the reaction mixture residue with high polar solvents (EtOAc and EtOH) the oily yellow oligomers were isolated.

The moderate yields of artemisinin are the result of a balance between factors stabilizing the artemisinin structure and the ease of side reactions. On the one hand, it was shown [80] that the strong anomeric n_O_ → σ*_CO_ interaction stabilizes artemisinin. On the other hand, in the peroxidation/cyclization step, highly reactive compounds can be easily oxidized, polymerized, and rearranged.

## 4. Conclusions

**Scheme 22:**
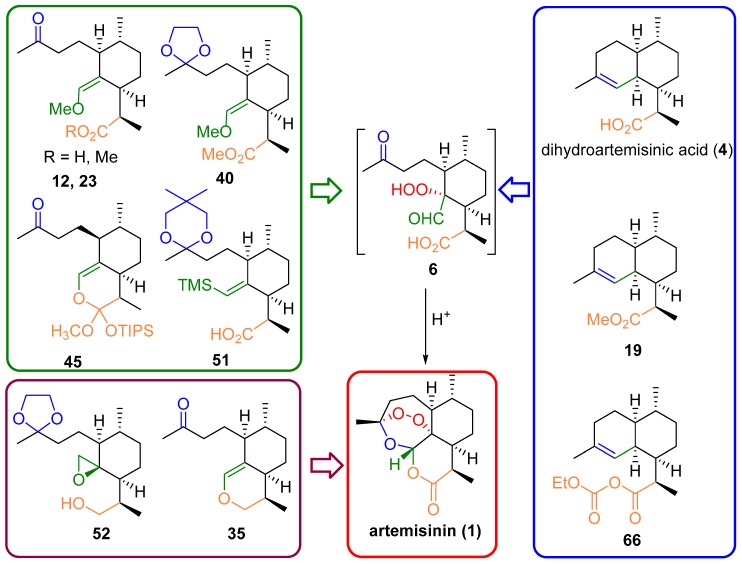
Summary of key-precursors in different approaches.

Analysis of the published data shows that synthetic approaches to the construction of peroxide ring in artemisinin has attracted increasing interest in recent times (Scheme 22). In total syntheses, various unsaturated keto derivatives **12**, **23**, **40**, **45**, or **51** were used as key precursors for the construction of artemisinin via hydroperoxyaldehyde **6**. The less common strategy lies in the construction of peroxide fragment based on precursors **35** or **52** followed by the subsequent formation of the artemisinin lactone cycle. In the past two decades, significant progress has been made in semi-synthetic methods. Compared with other routes to artemisinin, its semi-synthesis is still less studied, despite the fact that it could satisfy the demand for the cheap production of sufficient amounts of artemisinin. The semi-synthesis of artemisinin based on artemisinic acid seems to be more efficient and environmentally friendly because reliable sources are used in production and high-quality chemical methods (catalytic hydrogenation and photooxidation) are applied. Nowadays, artemisinin construction in semi-synthetic methods is performed through precursors **4**, **19**, or **66**.

The drawback of the majority of the available methods for the synthetic approaches to the artemisinin, which limits their application, is the low to moderate yield in the final peroxidation/cyclization step. In all published studies, the yield of artemisinin never exceeds 57%. Moreover, the synthesis often requires UV-irradiation equipment.

The main goals in the development of the synthetic strategies for the construction of a peroxide ring of artemisinin are as follows: (1) a development of methods for artemisinin precursor synthesis (artemisinic and dihydroartemisinic acids); (2) an investigation of the mechanisms of the peroxidation/cyclization step in order to predict optimal conditions for efficient synthesis; (3) the search for selective one-pot methods of peroxidation/cyclization; (4) applying green chemistry approaches to artemisinin production.

## Figures and Tables

**Table 1:**
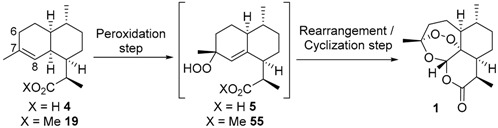
In situ transformation of dihydroartemisinic acid (**4**) or its ester **19** into artemisinin (**1**).

No.	Substrate	Condition of Peroxidation Step	Condition of Rearrangement/Cyclization Step	Yield of 1, %	Reference
1	**4**	O_2_/*hv*, Methylene Blue, acetone, 0 °C, 30 min	TFA (cat.), petroleum ether, air, r.t., 4 days	30	[59]
2	crude mixture of **19** and isomer with double bond C6-C7 position	O_2_/*hv*, Methylene Blue, CH_2_Cl_2_, 20 °C, 90 min	TFA (cat.), petroleum ether, air, 20 °C, 4 days	30	[64]
3	crude mixture of **4** and isomer with double bond C6-C7 position	O_2_/*hv*, Methylene Blue, acetone, 0 °C, 30 min	TFA (cat.), petroleum ether, air, 20 °C, 24 h	26	[65]
4	**19**	O_2_/*hv*, Rose bengal, CH_3_CN, −30 °C, 6 h	(1) O_2_, Cu(OTf)_2_, CH_3_CN, −20 °C;	25	[66]
			(2) *p*-TsOH (cat.), CH_2_Cl_2_, 20 °C, 4 h		
